# A life satisfaction approach to valuing the impact of health behaviours on subjective well-being

**DOI:** 10.1186/s12889-019-7896-5

**Published:** 2019-11-21

**Authors:** Yipu Shi, Craig Joyce, Ron Wall, Heather Orpana, Christina Bancej

**Affiliations:** 1 Centre for Surveillance and Applied Research, Public Health Agency of Canada, 785 Carling Ave. AL: 6809B, Ottawa, ON K1A 0K9 Canada; 2 Impact and Innovation Unit, Privy Council Office, Ottawa, Canada; 3 School of Epidemiology and Public Health, University of Ottawa, Ottawa, Canada; 4 Centre for Immunization and Respiratory Infectious Diseases, Public Health Agency of Canada, Ottawa, Canada

**Keywords:** Subjective well-being, Life satisfaction, Smoking, Physical activity, Fruit and vegetable consumption, Social impact assessment, Valuation, Social return on investment

## Abstract

**Background:**

Increasingly, decision-makers are interested in understanding the returns on investments in programs and policies that promote health and prevent chronic diseases. While the costs of these programs are more easily quantified, many of the outcomes they aspire to achieve are intangible and lack obvious market values. The subjective well-being (SWB) method was developed to value a wide range of non-market goods, including health outcomes directly in monetary terms. This paper presents an application of the SWB approach to estimate the monetary value of health-promoting behaviours as the intermediate outcomes of health promotion and chronic disease prevention programs and policies.

**Methods:**

Life satisfaction (LS) was used as a proxy of individuals’ SWB. Based on the combined Canadian Community Health Survey 2009–10 data, we modeled LS as a function of income and healthy behaviours, controlling for the socio-demographic factors associated with LS at the individual level using ordinary least squares regression. Equivalent effects of income and healthy behaviours on LS derived from the models allowed us to estimate the trade-off between income and healthy behaviours.

**Results:**

We found that income and healthy behaviours were positively associated with LS. The values of increased physical activity, an additional daily serving of fruits/vegetables, and not smoking are respectively $631, $115 and $563 per week. These represent the amounts of additional weekly income required to maintain an individual at their level of LS in the absence of each of these behaviours.

**Conclusions:**

The SWB method holds promise as a method to monetize the value of a range of non-market goods, including healthy behaviours for which market values do not exist. The SWB method can be applied efficiently and cost-effectively using readily available survey data.

## Background

Unhealthy behaviours such as physical inactivity, unhealthy diet, and cigarette smoking are common risk factors contributing to a wide range of chronic conditions such as obesity, diabetes, cardiovascular disease and cancer [1]. To save costs to the health care system and economy, governments make significant investments in the development of programs and policies that support the adoption of healthy behaviours [2]. These investments are believed to improve health related behaviours and outcomes, and are also likely associated with other individual or social benefits including increased educational attainment, employment opportunities, community engagement, and ultimately to an improvement of well-being. Because these kinds of behavioural outcomes usually lack obvious market values, they are often absent from economic burden and cost-benefit analysis, though a full impact assessment is needed for decision making.

Policy-makers are increasingly interested in understanding the returns on investments made in health-promoting policies and programs. There is a need for an approach to estimate the value of health outcomes in monetary terms to enable straightforward comparisons between the costs and benefits of investments in health-promoting programs. However, health behaviours frequently targeted by healthy living programs including healthy eating, physical activity, and smoke-free living do not have market values or existing market relationships that help infer monetary values, limiting further economic analysis to inform decision making or communicate impact to the public. Economists have at their disposal two established valuation techniques that have a long tradition in cost-benefit analysis [3]. The revealed preference approach relies on existing market relationships to implicitly derive values for non-market goods. For example, the trade-off between adverse health outcomes associated with certain jobs and the higher wages these jobs pay is a way to assign a market value to the level of risk workers assume [4]. The second established valuation approach is the contingent valuation technique, where a large sample of respondents is asked for their willingness to pay for a specific nonmarket good like good health or reduced crime [5]. Limitations to the contingent method include unreliable, unrealistic estimates or strategic behaviour when respondents are presented with situations with which they are unfamiliar [6, 7].

Recently, the subjective well-being (SWB) approach to valuation has emerged, and is used increasingly in the economic literature as a complement to the traditional valuation techniques in a wide range of policy contexts [3]. The SWB approach measures the actual trade-offs, but implicitly. It correlates the extent of nonmarket goods with individuals’ reported SWB, measured as a proxy of life satisfaction (LS), and evaluates them directly in relation to the effect of income on SWB. Using statistical modeling of SWB, it is possible to estimate trade-offs between income and nonmarket goods if SWB is kept constant. SWB is a broad concept that has been described as encompassing “Good mental states, including all of the various evaluations, positive and negative, that people make of their lives and the affective reactions of people to their experiences” [8]. LS is necessarily a subjective evaluation, which each individual makes based on his or her own criteria or standards [9]. The scores of LS contain information on the respondents’ global evaluation of their lives, with combined reflection of each individual’s stable inner state, current affect, present life and interpersonal comparability. Measures of LS, including single question measures of satisfaction with life, are well validated in the literature and have been incorporated into a number of national monitoring systems, including in Canada [8, 10]. Compared to traditional valuation methods, the LS method does not ask respondents to value the nonmarket good directly or make any trade-offs; instead it globally captures the factors that relate to how individuals evaluate their general LS. This is meant to off-set some of the weaknesses inherent to some traditional valuation methods, which often require respondents to imagine their behaviour in hypothetical situations, or fall prey to strategic behaviour in their responses. The LS approach has been widely applied to estimate the value of noise, air pollution, floods, crime and terrorism [11–13]. In the domain of health, it has been used to estimate the value of specific types of diseases (e.g. cardiovascular disease, cancer), mental health and health services [14–16].

In this study, we apply the LS methodology to value health promoting behaviours – physical activity, frequency of fruit and vegetable consumption, and smoke-free living that are currently the focus of healthy living programs funded by the Government of Canada. By applying the LS approach for the first time in a Canadian population health context, our analysis may lead to applications in other areas of policy to value intangible outcomes of investments that otherwise lack market values.

## Methods

### Data

The Canadian Community Health Survey (CCHS) provides individual-level self-reported data on LS, household income, health behaviours, as well as other LS associated socio-demographic variables identified in the economic literature. The CCHS is a national, cross-sectional survey administered by Statistics Canada that collects information on health status, health care utilization and health determinants of Canadians [17]. Data are collected from respondents aged 12 years and older living in private dwellings in the 10 provinces and 3 territories, covering about 98% of the Canadian population. The CCHS is conducted through telephone and in-person interviews. Informed consent is obtained from each participant and participation is voluntary. The overall response rate over the study period was 72.3%. In this analysis, we used Share File of the CCHS 2009–10, which contains data for 117,602 respondents who have agreed to share their personal data with Public Health Agency of Canada and Health Canada.

This study is a secondary analysis based on data from the CCHS conducted by Statistics Canada. All Statistics Canada surveys are subject to multiple stages of review by experts and senior management committees to address methodological, ethical and legal issues pertaining to data collection. Final approval of all surveys is required from Statistics Canada’s senior executives and the Chief Statistician of Canada, established by the *Statistics Act* to ensure the ethical conduct of data collection by Statistics Canada.

### Variables

In line with the well-being valuation literature we used life satisfaction (LS) as our measure of SWB. LS was measured using a single survey question: “Using a scale of 0 to 10 where 0 means “very dissatisfied” and 10 means “very satisfied”, how do you feel about your life as a whole right now?” Answers were coded numerically between 0 and 10. Scales such as this are necessary as research suggests that LS scales with at least seven choices capture 100% of the total variance in SWB [10].

Other variables identified in the economic literature as being associated with LS including income, education, employment status, marital status, health status, geographic area and home ownership were used to construct the LS model [18]. We used the individual’s best estimation of total annual household income before taxes and deductions of all household members from all sources in the past 12 months as our measure of income.

Health behaviour variables included leisure-time physical activity, frequency of fruit and vegetable consumption, and not smoking. Physical activity was measured based on the 1-month average frequency of all leisure-time physical activity sessions > 15 min that participants reported for a 3-month period. These responses were categorised into active (12 or more sessions per month); moderate (4 to 11 sessions per month); and inactive (less than 4 sessions per month). Frequency of fruit and vegetable consumption was measured as the number of times fruit and vegetables were consumed per day. Cigarette smoking was based on answers to the question: “At the present time, do you smoke cigarettes daily, occasionally, or not at all?” Respondents were categorised into non-smokers, occasional smokers, and daily smokers.

Self-perceived health status is a well-validated measure for assessing overall health. It correlates well with a number of health outcomes, including cardiovascular events, disability and mortality [19–21]. Self-perceived health status was captured by asking the question: “In general would you say your health is: excellent, very good, good, fair or poor?” Self-perceived mental health is a general assessment of the respondent’s own state of mental health, and focuses on aspects of health that are not necessarily reflected in self-perceived general health [22]. Respondents were asked to rate their mental health as excellent, very good, good, fair or poor.

Marital status was categorised as married, common-law, widowed, separated, divorced and single (never married). Other socio-demographic variables entered into the LS model included age (continuous), age-squared, sex (male/female), respondents’ educational attainment (<secondary, secondary graduation, some post-secondary, and post-secondary graduation), province or territory of residence, urban or rural residence, and home ownership (owned or rented). Employment status was defined as employed (full or part-time employed), student (full or part-time), retired (those responded “not applicable” to employment status and those aged 65 years and over), unemployed (those who responded “did not have a job last week”) and not in labour force (those who responded “permanently unable to work”). We also included immigration status, defined as being a recent immigrant (< 10 years in Canada), an established immigrant (10 years and more) or Canadian born.

### The LS valuation approach

The LS valuation approach was previously described by Ferrer-i-Carbonell and van Praag in its application to value chronic diseases [23]. Briefly, the LS approach assumes that LS of individual *i* is a linear function of the natural logarithm (ln) of household income (M), health-related behaviours (H) and other individual characteristics (X). The effects of these variables on LS can be estimated by an ordinary least squares (OLS) regression model based on eq. (1):

1 $$ L{S}_i=\alpha +\beta 1\ {M}_i+\beta 2\ {H}_i+\beta 3\ {X}_i+{\varepsilon}_i $$

where the β coefficients represent the proportional association of the logarithm of household income, healthy behaviours and other variables with LS, while ε is the measure of the error term. If the estimated coefficient for income is positive and significant, then based on the coefficients of income and healthy behaviours estimated from the OLS model, it is possible to approximate the equivalence between income and each of the healthy behaviours by estimating the increase in income that is required to offset the absence of each healthy behaviour in order to keep LS constant. The monetary value of a healthy behaviour can be calculated using eq. (2):

2 $$ \mathrm{Monetary}\ \mathrm{value}={\mathrm{M}}_0-{{{\mathrm{e}}^{\left[\ln \right(\mathrm{M}}}_0}^{\left)-\upbeta 2/\upbeta 1\right]} $$

where M_0_ represents the average annual household income of Canadians [24]. The proportional effects of income β1and healthy behaviours β2 on LS are taken respectively from eq. (1). The monetary value calculated for each of the healthy behaviour from eq. (2) was annual and was divided by 52 weeks to create a weekly amount.

### Statistical analysis

Continuous variables were inspected for normality and outliers using boxplots and descriptive statistics. Linear associations of LS with continuous variables including age, logarithm (income), and frequency of daily fruit and vegetable consumption were examined using scatterplots and inspection of fitted regression lines. The standard transformation to the natural logarithm for the income variable was conducted to account for the diminishing effect of income on LS [25]. Univariate analysis was performed to determine the variables significantly or borderline (*p* < 0.1) significantly associated with LS. Multivariable OLS regression models were constructed with the inclusion of variables identified in the univariate analysis. We assumed that our measure of LS was cardinal to allow for the use of OLS estimations that are straightforward to interpret, and also because it is commonly used in economic SWB literature [26]. Broadly, research in the area of SWB has not resulted in substantively different conclusions when SWB is modelled as an ordinal variable, as compared to when it is treated as interval and modelled using parametric statistics [27]. Variables that remained statistically significant and helped explain the variation in LS (assessed through the increase in adjusted R-squared values) were retained in the final model. The regression coefficients obtained for income and healthy behaviours were then used in eq. (2) to derive the trade-off values between income and the three healthy behaviours of interest.

### Indirect effect of income

Past research has suggested that income could exert an effect indirectly on LS through other variables with which income correlates [23, 28]. Not accounting for the indirect effect of income on LS through other variables in the model would result in the coefficient of income being underestimated, the consequence of which would be an overestimation of the value of the non-market goods of interest (in this case, healthy behaviours) [16, 29]. To account for this issue, we performed mediation analysis which estimated the indirect effect of income through other variables on LS in the model. These variables included self-perceived health, self-perceived mental health, and some socio-demographic variables. Operationally, this was done by removing each variable through which income may have an indirect effect on LS from the full model one at a time. The difference between the income coefficient obtained from these models and the full model is the indirect effect of income through that variable on LS. Aggregating the indirect effects of income from all mediating variables provides an estimate of the total indirect effect of income on LS.

### Sensitivity analysis

The robustness of the LS model constructed based on the CCHS 2009–10 data was tested by two sets of sensitivity analysis for model predictability and monetization variability, using 1) a different cycle of the CCHS (2005) and 2) a sub-population of seniors aged 65 and older from the original data used to construct the model.

To account for the complex multistage sampling design of the CCHS, we used 500 bootstrap weights created by Statistics Canada in our estimation of the variance for each of the parameter estimates. Statistical significance was assessed at *p* < 0.05. All analyses were performed using SAS 9.3 (SAS Institute Inc., Cary, NC) and SUDAAN 10.0.1 (RTI International Research Triangle Park, NC).

## Results

### Characteristics of the study population

Table 1 presents descriptive statistics of the study population. Nearly all (97%) of the survey respondents provided valid responses to the question on LS, resulting in a sample of 108,423 for the study. As income was used as a reference value in the LS model to which other variables were compared, individuals with missing values on household income were omitted to ensure biases were not introduced from imputation. Thus, the sample was further reduced to 74,682 for the analyses. Respondents with missing values on healthy behaviours and other socio-demographic variables were few and further exclusion resulted in a final sample of 74,577 records.

**Table 1:** Life satisfaction, household income, health behaviours, and selected socio-demographic factors of Canadians aged 12 years and older, Canadian Community Health Survey, 2009–10 (*n* = 74,577)

Dependent variable	Mean	95% Confidence Interval
Life satisfaction (0–10)	8.96	8.94–8.98
Explanatory variable		
Household Income (CAD)	71,975	71,236-72,713
Frequency of fruit and vegetable consumption (daily)	5.03	4.98–5.07
	Percentage (%)	95% Confidence Interval
Leisure time physical activity		
Active	27.4	26.8–27.9
Moderately active	25.4	24.9–25.9
Inactive	47.2	46.6–47.8
Type of smoker		
Daily	17.2	16.8–17.7
Occasionally	5.1	4.8–5.4
Not at all	77.7	77.2–78.1
Marital status		
Married	39.5	38.9–40.0
Common-law	10.2	9.9–10.5
Widowed	6.6	6.4–6.9
Separated	3.8	3.6–4.0
Divorced	8	7.7–8.3
Single	31.9	31.3–32.5
Self-perceived health		
Poor	2.5	2.4–2.7
Fair	8.6	8.3–8.9
Good	28.2	27.7–28.7
Very good	38.2	37.7–38.7
Excellent	22.5	21.9–22.9
Self-perceived mental health		
Poor	1.03	0.93–1.15
Fair	4.56	4.32–4.80
Good	20.7	20.3–21.2
Very good	36.9	36.4–37.5
Excellent	36.7	36.2–37.3
Home ownership		
Yes	67.8	67.1–68.5
No	32.2	31.5–32.9
Province/territory of residence		
Newfoundland and Labrador	1.52	1.47–1.58
Prince Edward Island	0.44	0.42–0.46
Nova Scotia	2.97	2.89–3.06
New Brunswick	2.37	2.30–2.43
Quebec	26.4	26.0–26.8
Ontario	36.1	35.7–36.5
Manitoba	3.55	3.43–3.67
Saskatchewan	2.87	2.79–2.96
Alberta	10.5	10.3–10.7
British Columbia	13.1	12.8–13.3
Territories	0.17	0.16–0.18

### Direct effect of income on LS

Multivariable OLS regression of LS on income, healthy behaviours and other variables explaining LS are presented in Table 2. As expected, ln (income) and healthy behaviours were significantly associated with LS. As well, some socio-demographic variables showed strong associations with LS. For example, we observed a clear dose-response association between self-perceived health, self-perceived mental health status and LS. Log-transformed income was linearly and significantly associated with LS, with each unit increase in the logarithm of annual household income increasing LS by 0.09 (95% confidence interval (CI): 0.06–0.11). This can be interpreted as the direct effect of income on LS (shown as β1 in eqs. 1 and 2). The healthy behaviours – physical activity, fruit and vegetable consumption and not smoking were positively associated with LS. It is noteworthy that when income was controlled, respondents who did not finish high school reported higher LS than those with post-secondary education in the model. Compared to those employed full-time and part-time, respondents not in the labour force were significantly less satisfied with their lives (β = − 0.23, 95% CI: − 0.37, − 0.10). However, when analysis was stratified by gender, significantly lower LS was seen only in men (not in labour force: β = − 0.33, *p* < 0.001; not employed: β = − 0.09, *p* = 0.026), but not women (β = − 0.15, *p* = 0.14; β = 0.008, *p* = 0.81) in comparison to employed men and women (data not shown).

**Table 2:** A life satisfaction model for Canadians aged 12 years and old, Canadian Community Health Survey, 2009–10

	Coefficient^*^	95% Confidence interval
Explanatory variable		
Constant	9.45	9.13, 9.76
Logarithm (household income)	**0.09**	**0.06, 0.11**
Health Behaviours		
Leisure time physical activity		
Active	**0.14**	**0.12,0.21**
Moderately active	**0.08**	**0.05,0.16**
Inactive	Reference	
Frequency of Fruit and vegetable consumption (daily)	**0.02**	**0.01,0.02**
Type of smoker		
Daily	Reference	
Occasionally	0.07	−0.01,0.15
Not at all	**0.12**	**0.08,0.17**
Socio-demographic variable		
Age	**−0.03**	**− 0.04,-0.02**
Age^2^	**0.0003**	**0.0002, 0.0004**
Sex		
Male	**−0.12**	**− 0.15,-0.09**
Female	Reference	
Marital status		
Married	Reference	
Common law	−0.04	−0.09,0.01
Widowed	**−0.33**	**− 0.40,-0.25**
Separated	**−0.48**	**−0.58,-0.38**
Divorced	**−0.26**	**−0.33,-0.20**
Single	**−0.32**	**−0.37,-0.28**
Education		
Less than secondary	**0.1**	**0.05,0.15**
Secondary	0.04	−0.00,0.08
Other post-secondary	−0.04	−0.11,0.03
Post-secondary	Reference	
Perceived health status		
Poor	**−2.19**	**−2.35,-2.02**
Fair	**−1.15**	**−1.22,-1.07**
Good	**−0.59**	**−0.63,-0.54**
Very good	**−0.30**	**− 0.34,-0.26**
Excellent	Reference	
Perceived mental health status		
Poor	**−3.13**	**−3.43,-2.83**
Fair	**−1.91**	**−2.00,-1.81**
Good	**−1.01**	**−1.06,-0.96**
Very good	**−0.47**	**−0.50,-0.44**
Excellent	Reference	
Employment status		
Employed	Reference	
Retired	0.05	−0.03,0.13
Students	0.05	−0.04,0.13
Unemployed	−0.03	−0.08,0.03
Not in labour force	**−0.23**	**−0.37,-0.10**
Immigration status		
Recent	**−0.18**	**−0.26,-0.10**
Established	**−0.14**	**−0.20,-0.09**
Canadian born	Reference	
Home ownership		
Yes	Reference	
No	**−0.11**	**−0.16,-0.07**
Urban/rural residence		
Urban	Reference	
Rural	**0.1**	**0.07,0.14**
Province/Territory of residence		
Newfoundland and Labrador	**0.23**	**0.16,0.31**
Prince Edward Island	**0.23**	**0.12,0.34**
Nova Scotia	**0.16**	**0.09,0.24**
New Brunswick	**0.28**	**0.22,0.35**
Quebec	**0.12**	**0.07,0.16**
Ontario	Reference	
Manitoba	**0.1**	**0.02,0.18**
Saskatchewan	**0.19**	**0.12,0.25**
Alberta	0.02	−0.03,0.08
British Columbia	0.01	−0.03,0.06
Territories	**0.16**	**0.04,0.28**
Number of participants	74,577	
Overall R^2^	0.3302	

The life satisfaction model was constructed using ordinary least squares regression with income, health behaviours and socio-demographic variables included in the model

*Coefficient (bold): Statistically significant (*p* < 0.05)

### Indirect effects of income on LS

Table 3 presents the results of the mediation analysis, demonstrating the indirect effects of income through other variables on LS. We found that income has a positive indirect impact on LS through marital status, perceived health, perceived mental health and home ownership, as evidenced by increases in the income coefficient from 0.09 to 0.14, 0.13, 0.12 and 0.11, respectively when each of these mediating variables was removed from the model. Variables including age, sex, education, employment status and length of immigration were not removed in the mediation analysis because income cannot change age and sex and it is unlikely to affect education, employment and the length of immigration. The cumulative overall effect of dropping each of the variables was an increase in the income coefficient by 0.14. This is the aggregated indirect effect of income. It was added to the direct effect of income (0.09) from the full model, leading to a total effect of income of 0.23. The total income coefficient of 0.23 is the β_1_ value that is used in the calculation of the adjusted monetary value in Eq. (2).

**Table 3:** Indirect effects of income through other variables on life satisfaction

	Income coefficient (95% CI)	Change in income coefficient
Full model	0.09 (0.06,0.11)^a^	
Variable dropped^b^		
Mental health status	0.13 (0.10, 0.15)	0.04
Health status	0.12 (0.10, 0.15)	0.03
Marital status	0.14 (0.12, 0.16)	0.05
Urban/rural residence	0.08 (0.06, 0.11)	−0.01
Home ownership	0.11 (0.09, 0.13)	0.04
Province of residence	0.08 (0.06, 0.10)	−0.01
Total indirect effect		0.14

Indirect effect of income on life satisfaction was analysed by mediation analysis

^a^Income coefficient was obtained from the full life satisfaction model in Table 2

^b^Income coefficient was obtained after one variable was dropped from the full model through mediation analysis

### Monetary valuation of health-related behaviours

Table 4 shows the monetary valuation of healthy behaviours with and without adjustment for the indirect effect of income. It can be interpreted as the extra income required, in absence of a specific healthy behaviour, to produce the same level of LS as if he or she were physically active, ate an additional serving of fruits and vegetables, or did not smoke. By accounting for the indirect effect of income, estimated monetary values on average fell by nearly 50%. Based on the SWB valuation framework, these are values in addition to any market prices paid to assist behavioural changes such as the costs of fitness equipment or smoking cessation aids.

**Table 4:** Comparisons of monetary values of health behaviours estimated based on income with/without indirect effect adjustment

Variables	Direct effect of income		Full (direct and indirect) effect of income	
	β Coefficient	Monetary value^c^	β Coefficient	Monetary value
Logarithm (household income)	**0.09**^**a**^	n/a	**0.23**^**b**^	n/a
Leisure time physical activity				
Active	0.14	$1092/week	0.14	$631/week
Moderately active	0.08	$815/week	0.08	$407/week
Frequency of fruit and vegetable consumption (daily)	0.02	$276/week	0.02	$115/week
Smoke-free	0.12	$1019/week	0.12	$563/week

^a^Income coefficient was obtained from the full life satisfaction model in Table 2

^b^Income coefficient was the sum of direct (Table 2) and indirect (Table 3) effect of income

^c^Monetary value was estimated using the formula: M^0^ – e^[ln(M0)- β2/ β1]^

M^0^: Annual average household income: $71,975; β1: coefficient of income (Bold); β2: coefficient of health behaviours

The results of sensitivity analysis are presented in Table 5. We repeated the same analyses using data from the CCHS 2005 and found that the direction of the associations between income, healthy behaviours and other socio-demographic variables and LS persisted, although the magnitude of the effect of income on LS decreased. The overall predictive capacity of the model remained comparable (R^2^: 0.30 with 2005 data vs. 0.33 with 2009–10 data). The variability in model prediction between years could be due to the residual effect of unknown or unmeasured time-varying factors related to LS. Time-dependent factors such as macroeconomic factors have been evidenced to have an impact on health and well-being [30]. For example, the 2008 global financial crisis [31] could have been a factor impacting Canadians’ SWB over time.

**Table 5:** Sensitivity analysis (Model robustness was checked using different data sets)

Variables	Model 1 (original model)			Model 2 (test data 1)			Model 3 (test data 2)		
	Coefficient	SE	Adj. R2	Coefficient	SE	Adj. R2	Coefficient	SE	Adj. R2
Logrithm (income)	0.09	0.03		0.05	0.01		0.11	0.03	
Leisure time physical activity									
Active	0.16	0.02		0.08	0.01		0.18	0.05	
Moderately active	0.11	0.03		0.05	0.01		0.08	0.06	
Frequency of fruit and vegetable consumption (daily)	0.02	0		0.01	0		0.02	0.01	
Smoke-free	0.12	0.02	0.33	0.06	0.01	0.299	0.22	0.06	0.2723

Model 1: Canadian Community Health Survey 2009–10

Model 2: Canadian Community Health Survey 2005

Model 3: Canadian Community Health Survey 2009–10 (ages > 65 years)

All variables were statistically significant (*p* < 0.05)

Seniors are at a distinct stage of the life course, and may present a different perspective on SWB with respect to income, healthy behaviours and other factors due to their changing social roles and perspectives. We found that the direction of the association of income, healthy behaviours and other variables with LS still held, but that the magnitude of the associations with these variables became larger, suggesting that perhaps as people age, health, healthy behaviours and income tend to relate more to one’s well-being than the general population. The overall predictive power of the model decreased slightly (R^2^: 0.27 vs. 0.33) suggesting other factors pertinent to LS among seniors may need to be explored.

## Discussion

This study applied the SWB valuation method to monetize healthy behaviours including physical activity, frequency of fruit and vegetable consumption, smoke free living, all of which are routinely collected in the health surveys. We found that healthy behaviours - physical activity, frequency of fruit and vegetable consumption and smoke free living were positively associated with the SWB of Canadians. Using the SWB valuation method, the monetary values calculated for these behaviours represent the additional income required to bring someone who does not exhibit a particular healthy behaviour to the same level of SWB as someone who does exhibit that behaviour. Incorporating mediation analysis, the method improves our ability to take into account the full direct and indirect effects of income on LS, allowing estimation of a more realistic monetary value for healthy behaviours of interest. The LS approach to valuation can be used in many areas of policy as a useful alternative to traditional valuation techniques. This method shows promise in providing decision-makers, who need to choose from among a suite of social and economic programs, with a comparable metric to value program impacts that may not have market values.

The estimation of the β coefficient of income in the regression model is critical as all monetary values derived in relation to the full causal effect of income on LS. This has been especially challenging for income because other variables that drive LS may also correlate with income within the model. Controlling for variables that partially rely on income to have effects on LS would remove these effects from the full effect of income, resulting in smaller β coefficient of income variable and excessive monetary values of nonmarket goods. Recent economic studies have attempted to account for the indirect effect of income using mediation analysis, in which the values for employment and the cost of the burden of being in debt fell by one-half when adjustment was applied [29]. Although our study has controlled for as many of the determinants of LS as possible in our regression models, our inability to include all variables related to LS prevents the full causal effect of income on LS from being fully accounted for with our approach. However, our method presents a useful way of achieving a more accurate and realistic valuation of nonmarket goods for decision making. To further deal with the issue of causality and the full effect of income in LS models, recent economic studies have attempted to use an instrumental variable (IV) approach, which eliminates the correlation between the income variable and the error term of the LS model. For example, using lottery wins as the IV, a study commissioned for the UK government estimated the well-being impacts of culture and sport [24, 32]. The β coefficient of the income variable in the LS model was estimated at 1.16 for the UK population, which is almost five times that of our estimate (0.23) and considered the causal effect of income on LS. With further studies adopting the IV approach to verify the true effect of income on LS in Canadian population, we anticipate that the monetary values of healthy behaviours estimated in this study will decrease substantially if the causal effect of income on LS is fully accounted for.

A key strength of this study is the analysis of routinely available, large-scale population survey data collected, thus overcoming the major weaknesses inherent to the traditional valuation methods (unreliable and strategic biases). Furthermore, the survey contains a rich source of socio-demographic and microeconomic variables on a representative sample of the population which would not be possible for most traditional valuation methods, thus allowing for the control of as many of the determinants of LS as possible using regression analysis. Lastly, our study accounted for the indirect effects using the mediation approach, which is a methodological strength over the majority of valuation studies using the LS approach in the literature. By accounting for the indirect effect of income, estimated monetary values in our study on average fell by nearly 50%, consistent with the economic literature which estimated monetary values are almost two to three times as large as valuation based on an uncorrected income coefficient [29].

Several limitations of this study should be noted. First, the data used in this study are cross-sectional, thus reverse causation is always a possibility with this type of data [3]. In addition to income and healthy behaviours of interest, other socio-demographic variables (employment status, house ownership and place of residence) and health status (self-reported health and mental health) were significantly associated with LS in the model. The causal effects of these factors on LS cannot be determined in the current study. Inconsistent findings using cross-sectional and longitudinal data was well illustrated by a Finnish study examining the effect of unemployment on self-reported health [33]. An experimental approach observing changes in well-being before and after interventions targeting healthy behaviours would be interesting to explore. Second, we believe the LS model for valuing healthy behaviours constructed in this study contains the main determinants of LS as indicated by an R-squared value of 0.33, which is higher than other models developed for a similar purpose in the literature. Nonetheless, we acknowledge that unobserved factors that do not change over time influence LS cannot be examined with cross-sectional data. Future studies using longitudinal data to control for unobserved time-invariant factors through the use of fixed effects regression analysis could further improve the model’s predictive power. Third, approximately 30% of respondents had missing values on the income variable, which may bias our estimation. As income is a key variable and the effect of income on LS serves as a reference value with which other variables will be compared, we decided not to use imputation for missing values on income. However, the reliability of the income variable could be improved by linking surveys with tax data in the near future. Fourth, some determinants of LS such as health status and mental health status are subjectively measured and may be a source of shared methods variance. Future studies incorporating objectively measured functional health into the LS model may be desirable. Finally, while we examined the stability of the model across one sub-population, adults aged 65 years and older, it would be critical to conduct subsequent sensitivity analyses with other subpopulations, such as men or women, or for those who are employed, unemployed, or out of the labour force.

## Conclusions

This study has introduced the LS approach to value healthy behaviours that evaluators can use to assess the returns on investments in health promotion and chronic disease prevention programs. The LS method is a promising approach to value otherwise intangible policy and program outcomes using readily available survey data at a low cost, and can support decision-making based on a common and interpretable metric. From a policy perspective, there is growing acknowledgment that wellbeing analysis could become a new standard of practice as part of a new generation of cost-benefit analysis [34]. In addition, it offers insight into the unfiltered preferences of communities of people and this creates the potential to identify new policy priorities that may otherwise be overlooked by policy makers [35]. Future research is needed to continue to understand the optimal use of this approach across various behaviours and in relation to other valuation techniques.

## Data Availability

The data that support the findings of this study are available from Research Data Centres situated in Statistics Canada and secure university settings across Canada. Access to public use microdata of CCHS is offered free of charge and the Population Health Surveys Data Access Unit in Statistics Canada (cchs-escc@statcan.gc.ca) can be contacted for more information.

## Abbreviations

CCHS: Canadian Community Health Survey;
IV: Instrumental variable
LS: Life satisfaction
OLS: Ordinary least squares
SWB: Subjective well-being
